# The Big Bang: How Does Indoor Noise Affect Lifestyle Choices of Older Community‐Dwelling Adults? An Exploratory Mixed Methods Study

**DOI:** 10.1111/ajag.70226

**Published:** 2026-09-07

**Authors:** Alison Short, Danielle Ní Chróinín, Heavenlia Rajendran, Christopher Poulos, Anne Hogden

**Affiliations:** ^1^ Faculty of Medicine and Health, UNSW Sydney Sydney New South Wales Australia; ^2^ School of Humanities and Communication Arts, Western Sydney University Sydney New South Wales Australia; ^3^ Department of Geriatric Medicine Liverpool Hospital Liverpool New South Wales Australia; ^4^ South Western Sydney Clinical School, UNSW Sydney Liverpool New South Wales Australia; ^5^ Centre for Positive Ageing, HammondCare Hammondville New South Wales Australia

**Keywords:** aged, hearing, lifestyle, noise, patient outcome assessment

## Abstract

**Objectives:**

To examine self‐reported noise sensitivity and how older community‐dwelling adults subjectively experience noise in community‐based venues encountered in day‐to‐day life.

**Methods:**

This exploratory mixed method study included older adults residing in an independent living community in New South Wales, Australia. It comprised a voluntary anonymised paper‐based survey that included questions/statements from the Noise Sensitivity Scale (3‐NS) and Noise Sensitivity Questionnaire (NoiSeQ), plus four focus groups exploring participants' experience of indoor auditory environments.

**Results:**

Participants (*n* = 50) reported a moderately high sensitivity to noise (median 9/15 on 3‐NS, IQR 8–10). Those with hearing aids (23/50) were more likely to score higher in total sum of NoiSeQ questions (OR 1.15, 95% CI 1.01–1.30, *p* = 0.03). Shopping venues (32/50), food courts (32/50) and clubs (24/50) were commonly identified as noisy environments. Key themes identified in focus groups (*n* = 20) encompassed: (1) ‘noise’ versus ‘music’; (2) acceptance of sound; and (3) coping strategies. Factors affecting the perception of sound as ‘noise’ included the self‐perceived health of the listener, and qualities, familiarity and perceived necessity of the sound. Older adults employed a range of coping strategies, including adjustment of hearing aids and avoidance strategies, such as online shopping and shopping at non‐peak times.

**Conclusions:**

Older adults may be highly sensitive to indoor noise and demonstrate a range of coping strategies to deal with noise in community‐based venues. Greater engagement with consumers to determine their noise‐related needs is necessary in order to support ongoing lifestyle activity and engagement in the community.

## Impact Statements

In a mixed methods study including 50 community‐dwelling older people, unwanted noise was reported to be common, affecting experiences of community venues and requiring a range of coping mechanisms. Our findings have implications for environmental noise policies and marketing strategies employed by community providers to better cater for and support the older adult population.

## 1 Introduction

The focus of healthy ageing within a public health approach seeks to promote full participation of older adults within their communities by maintaining and enhancing the individual's intrinsic capacity as they age [1]. Community indoor environments are a crucial part of accessible everyday activities, providing opportunities for both physical activity and social engagement. Social participation, such as leisure, social, cultural and spiritual activities in the community, is strongly associated with good health and well‐being throughout life, including into older age [2, 3]. Social participation is associated with positive benefits on health [3], including maintenance or establishment of supportive and caring relationships, continued competence and enjoyment of respect and esteem [2]. Social disengagement is associated with age‐related cognitive decline, whereas more stimulating activities may be associated with cognitive preservation [4]. However, as people age, engagement in these activities typically declines [5], suggesting possibilities to improve older adults' health via encouraging greater community participation in line with the social determinants of health [2]. Factors that affect healthy lifestyle choices and engagement by community‐dwelling older adults include, amongst others, knowledge and awareness of events [2, 6], affordability, accessibility [2, 7, 8], a desire to stay active [9] and noise. The negative impact of noisy acoustic environments on the mental health of older adults has been well documented [10], and the cognitive‐motor interference of excessive noise may affect mobility [11] and contribute to distractibility [12], influencing falls risk.

Environmental noise is considered to be an ongoing public health problem [13]. Noise is defined now primarily as unwanted sound [14, 15], ‘a pollutant whose effects on health have been neglected’ [15, p. 2] with Amman and colleagues noting that ‘irrespective of the causation, uncontrollable noise exposure is unpleasant and affects the quality of life’ [16, p. 1]. Negative effects of excessive noise on human health include cardiovascular disease, metabolic disease, cancer and respiratory disease [17, 18]. Noise is not simply a physical, but also a perceived phenomenon, relating to the individually variable experience of sound, and noise exposure is understood to also affect the central nervous system and mental health via a sustained stress response pathway [18]. The nature and level of how noise is ‘unwanted’ changes with experiences, activities and goals, the physiological state and needs of the individual [19]. Noise can impair the ability to enjoy one's leisure time and increases the frequency of negative or anti‐social behaviour, such as aggression, unfriendliness, non‐participation or disengagement [14]. Noise adversely affects general health and well‐being in the same way as chronic stress [14]. Noise annoyance is regarded as one of the most pervasive negative effects of environmental noise exposure [20]. Annoyance due to environmental noise is influenced by several non‐acoustic factors, such as personal traits and attitudes towards the source [21, 22]. Noise sensitivity acknowledges that individuals vary in reactivity to noise [16], and significantly influences the annoyance level caused by both indoor and outdoor noise [22, 23]. Around a third of surveyed populations will judge themselves to be noise sensitive [16]. Furthermore, noise—especially excessive and/or prolonged—may contribute to hearing loss, adversely affecting social interactions and cognitive performance, and potentially mental health [24].

A range of studies have explored how people experience and react to noise, particularly in occupational settings [25, 26, 27], and outdoor environments, such as with aviation noise or traffic [24, 28, 29]. However, noise within the indoor environment has been less studied, despite the existence of indoor acoustic design standards [30] which also cover indoor experiences of externally generated noises. In fact, much of everyday life for older adults includes indoor environments. Within the hospital environment, excessive noise causing noise stress has been well documented [31, 32, 33], and combined noise sensitivity and mental health effects of noise in dining/restaurant experiences have been explored [34, 35]. Within the published noise studies, few have included older adults, and none have focused solely on their community‐dwelling experiences, although a handful of studies have investigated the potential association between noise and the behavioural and psychological symptoms of dementia [36].

Anecdotal evidence suggests that the auditory environment has an influence on lifestyle choices, and certain activity locations may be avoided by older people due to high noise levels [2]. However, to date, there has been a dearth of evidence regarding the perceptions and effect of the auditory environment on older adults living in the community, including in supported living environments, and how these might affect their lifestyle choices, in turn potentially impacting their physical activity and social engagement.

Our aim was to explore how older people subjectively experience noise and self‐report their sensitivity to noise. Based on previous results [31, 35], we hypothesised that older adults would be sensitive to noise and that community environments perceived as ‘noisy’ may lead to avoidance behaviours. For this study, older adults situated in independent living units (ILUs) within a larger aged care development were targeted as they regularly accessed the broader (external) community for shopping, social activities and other lifestyle engagements. Such ILUs are typically small cottage‐type low density buildings designed for a single household, typically situated within a retirement community [37].

## 2 Methods

We conducted a mixed methods explanatory sequential research design study [38] comprising a self‐report survey followed by optional focus groups with older adults residing in an independent living community in metropolitan Sydney, New South Wales, Australia, in mid‐2014. Within the local urban area, many community facilities were available, including small and large shopping centres and a range of cafe, restaurant and club environments. All residents of an independent living aged care community (total *n* = 122) were eligible for the study provided they were aged 60 years or older and could understand English.

Our paper‐based survey (Appendix S1) included assessment of self‐reported noise sensitivity, and of the impact of noise. Where possible, modifications to existing tools were introduced to (a) focus on our aims, (b) adapt to consumer preference and (c) reduce the burden of multiple questions. Initial drafts of the survey were reviewed by two consumers and three staff members accustomed to working closely with the residents.

Our sensitivity rating was derived from the Noise Sensitivity Scale (3‐NS), based on the tool developed and validated by Amann et al. [16], with three statements constructed to investigate vulnerability to noise, sensitivity to noise and self‐rated coping with noise, using a Likert scale. We asked participants to rate the three statements from strongly disagree (1) to strongly agree (5), with a total score based on the sum of individual questions (Appendix 1: Survey [section 2, 1–3]) [16]. The 3‐NS was developed by Amman et al. [16] in order to strike a balance between more lengthy questionnaires and single‐question tools. We chose this tool because it is brief, easy to understand, of limited research burden, and easy to complete as a participant self‐rating Likert scale.

To determine the impact of noise on activities undertaken, we utilised 18 questions from the NoiSeQ, originally developed and validated by Schutte et al. [39, 40] (Supporting Methods and Appendix S1: Survey [section 3]). The NoiSeQ measures ‘global noise sensitivity as well as the sensitivity of five domains of daily life, namely, leisure, work, habitation, communication, and sleep’ [40, p. 15]. Modifications were made to the NoiseQ following stakeholder consultation (Supporting Methods).

We additionally gathered participant characteristics: gender, age, previous occupation, sensory and mobility deficits, pain and other conditions affecting participation in daily activities.

Culturally and linguistically diverse countries were defined as those other than Australia, New Zealand, United Kingdom, Ireland, United States of America, Canada and South Africa [41]. In addition, we provided a non‐exhaustive list of potentially noisy places, generated in consultation with stakeholders, that respondents might consider noisy, along with an ‘other‐ please specify’ option. ‘Clubs’, although not strictly defined for purposes of this survey, in Australia were generally understood to be social venues, such as the Returned and Services League of Australia, which provide food, beverages and entertainment for the community [42].

The anonymous hard‐copy (paper‐only) survey was distributed to residents' usual mailing systems, flyers and collection containers strategically placed in common areas for ease of return. Residents were able to indicate interest in participating in focus groups at the end of the survey, separate to surveys, to maintain anonymity. The semi‐structured interview guide for the focus groups was designed to address our aims and informed by a relevant literature review (Supporting Methods). Focus groups were digitally recorded and transcribed.

### 2.1 Quantitative Data Compilation and Analysis

Quantitative data from the surveys were entered electronically into Microsoft Excel and analysed using descriptive statistics. Questions were separated into 3‐NS scores and NoiSeQ scores, then analysed as follows: per previous studies [43], and responses inverted as needed to align with outcome of (positive) noise sensitivity (Supporting Methods).

Associations between hearing impairment and aids and noise sensitivity were explored using simple comparisons and basic regression analysis to explore factors (age, gender, former occupation and use of hearing aids) and noise sensitivity. We allowed for potential multivariable regression analysis depending on total numbers and strength of association from univariate analysis.

### 2.2 Focus Group Data and Analysis

Four focus groups (labelled A–D) were facilitated by authors AS and HR, allowing flexibility in participant attendance; there was no specific number limit to the number of participants per group. Participants were provided with numbered badges to enable deidentification within the recorded transcript. Participant quotes are therefore labelled with a letter and number. Thematic analysis was conducted whereby textual data were chunked into separate idea units and tagged with codes [44]. Each code consisted of a summarised paraphrase of the idea. Thematic categories were then created to group codes of similar ideas. Similar thematic categories were then grouped together under a higher order ‘key theme’, with main themes falling below this, and subthemes below this again. A lengthy iterative process was undertaken which involved checking thematic codes against the original data to ensure that they had adequately captured the meaning of the idea units. To optimise rigour and trustworthiness, semi‐structured questioning techniques were employed during focus groups, with on‐the‐spot member checking and tactics directed at frank discussion. A clear audit trail was used to keep track of the decision‐making processes, and reflective field notes were reviewed and discussed with two supervisors.

### 2.3 Ethics Statement

This study was approved by the University for New South Human Research Ethics Advisory Panel (9_13_044) and all participants provided written informed consent.

## 3 Results

Amongst all 122 residents, 50 residents completed the survey (41% response rate; 50/122); characteristics are shown in Table 1. The commonest former careers were more‐than‐one industry (*n* = 11), administrative (*n* = 7) or home duties (*n* = 5).

**TABLE 1:** Patient characteristics based on respondent survey responses (*n* = 50).

Patient characteristic	Distribution
Age, mean, SD (range)	79.2, 8.41 (63–95)
Gender, *n* (%)	
Female	33 (66)
Male	15 (30)
Undeclared	2 (4)
Country of birth, *n* (%)	
Australia	45 (90)
CALD countries	3 (6)
Other non‐CALD countries	2 (4)
Select comorbidities^a^, *n* (%)	
Hearing impairment	37 (74)
Reported as severe/profound	7 (14)
Visual impairment	49 (98)
Reported as severe^b^	3 (6)
Requiring corrective lenses	49 (98)
Mobility impairment, *n* (%)	27 (54)
Reported as severe^b^	4 (8)
Requiring mobility aid	13 (26)
Previous fall requiring hospitalisation, *n* (%)	30 (60)
Chronic pain, *n* (%) Reported as severe^b^	4 (8)
Other problems reported to affect daily activities^c^	7 (14)
Use of hearing aids, *n* (%)	23 (46)
Bilateral	19 (38)
Unilateral	4 (8)
Previous occupation, *n*	
> 1 area/industry	11
Academic	1
Administrative	1
Business owner	1
Construction	1
Education	1
Finance	1
Healthcare	3
Home duties	5
Hospitality	1
Law enforcement/security	2
Manufacturing	3
Religious/missionary	3
Retail	2
Not specified	9

^a^ Comorbidities which were selected as potentially impacting on engagement with activities in community‐based locations.

^b^ Nil profound.

^c^ Free text responses, including arthritis (*n* = 3), heart failure (1), dyspnoea on exertion (1), knee replacements (1) and prostate‐related incontinence (1).

### 3.1 Noise Sensitivity

**FIGURE 1:**
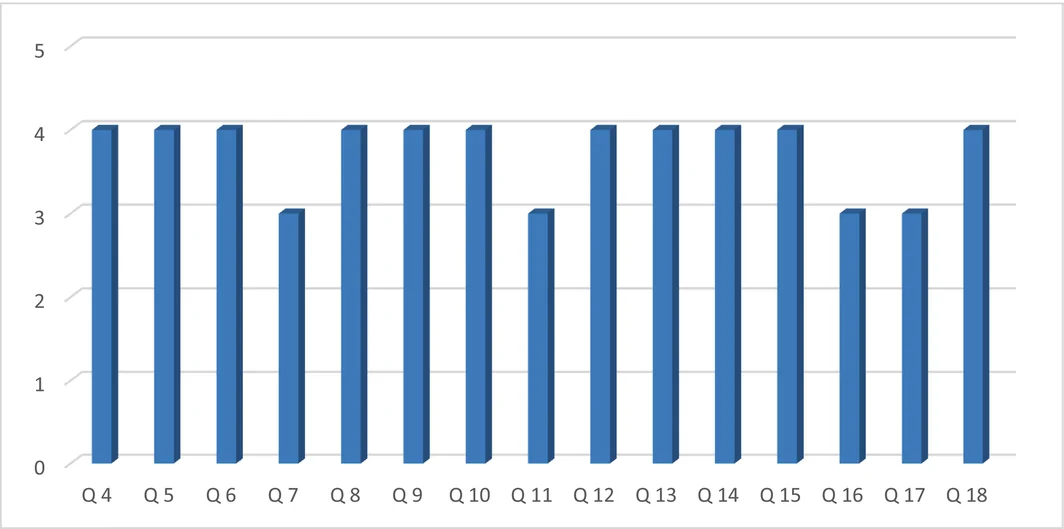
Median Likert scores for questions taken from the NoiseQ tool (Questions 4–18 on our questionnaire, Appendix S1). Please see also Table S1. Likert scores for Questions 7, 8, 11 and 16 were inverted to align with ‘positive’ noise sensitivity.

Three survey questions assessed noise sensitivity, where an elevated score indicated higher sensitivity to noise. Median score (no missing data) was 3 (IQR 2–4) for each of three 3‐NS statements: ‘I think I am less sensitive to noise that others’, ‘I think I can cope with noise better than others’, and ‘I often feel completely overwhelmed by noise’ (inverted scoring for the latter question). The total median score for all three questions was 9 (8–10), range 4–14, indicating moderate noise sensitivity amongst our respondents. Median scores for questions adapted from the NoiSeQ are shown in Figure 1; 10/150 responses were left blank. Median scores for individual questions indicated that respondents often reported agreement with statements indicating everyday noise sensitivity across a broad range of scenarios.

### 3.2 Factors Potentially Associated With Noise Sensitivity

Participants with hearing aids (*n* = 23) were more likely to score higher using the NoiSeQ questions total score (OR 1.15, 95% CI 1.01–1.30, *p* = 0.03), but not using the 3‐NS (OR 0.92, 95% CI 0.67–1.26, *p* = 0.61). Neither age (correlation coefficient 0.22, 95% CI −1.17 to 1.61, *p* = 0.76) nor gender (OR for male 0.90, 95% CI 0.64–1.28, *p* = 0.56) were associated with noise sensitivity as assessed by 3‐NS. Likewise, no association was seen between total NoiSeQ questions score and age (correlation coefficient 0.11, 95% CI −0.29 to 0.51, *p* = 0.58) or male gender (OR 1.05, 95% CI 0.94–1.17, *p* = 0.4). Given the small numbers in any category of former occupation, we did not analyse for association between this and noise sensitivity.

### 3.3 Noisy Community Locations

**FIGURE 2:**
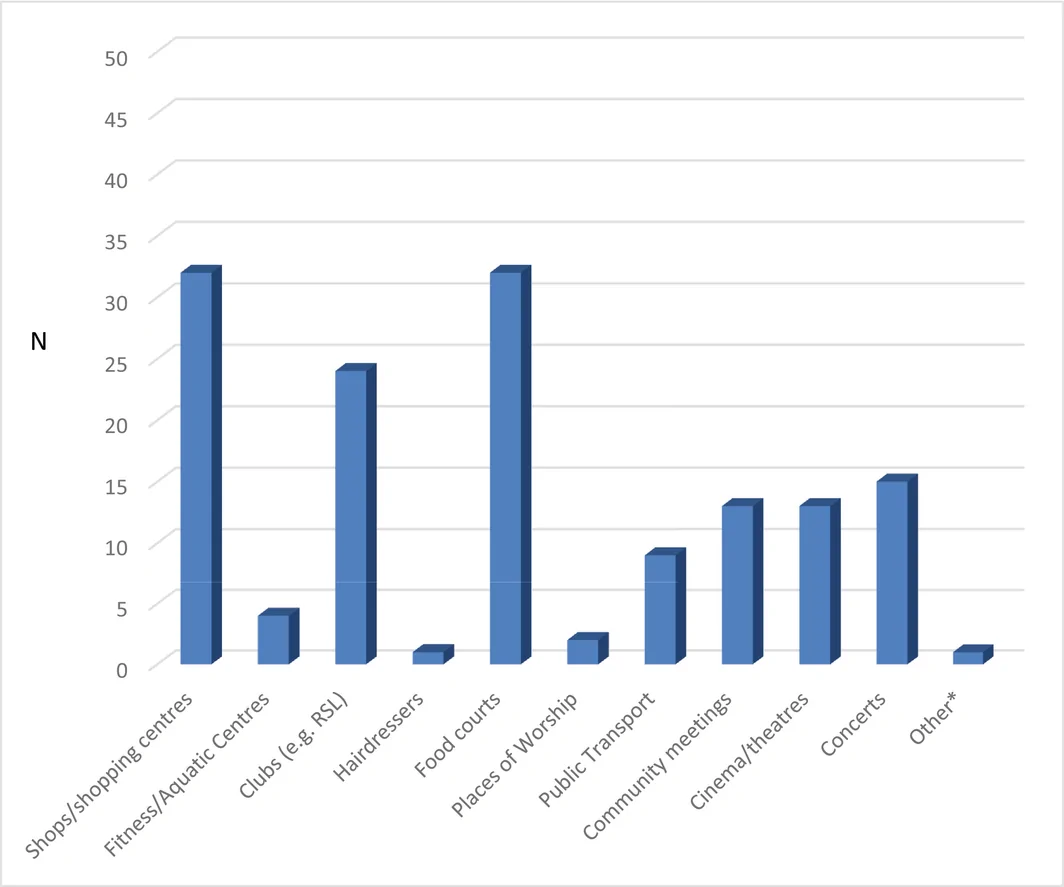
Places in the community reported as noisy by respondents (total respondents *n* = 50). Respondents could include more than one location. Clubs were not strictly defined, but are usually understood here in Australia to be social venues, such as the Returned and Services League of Australia, which provide food, beverages (including, usually, a liquor licence) and entertainment for the community. Other (*n* = 1) specified by the respondent as ‘city footpaths and traffic’.

When asked about indoor community locations that were very noisy, shopping venues (64%), food courts (64%) and clubs (48%) commonly were identified as ‘very noisy’ (Figure 2). Hairdressers (2%) and places of worship (1%) were less often reported as noisy.

### 3.4 Focus Groups

**FIGURE 3:**
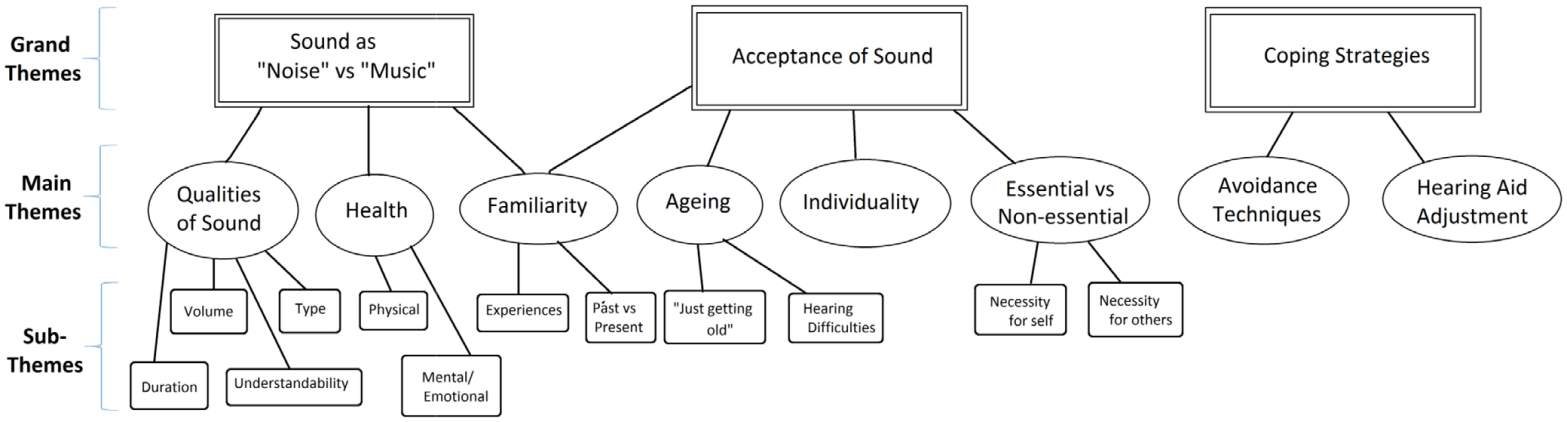
Themes identified from qualitative data from focus groups (total *n* = 20 participants).

Four focus groups were conducted with 20 participants who had completed the survey, with four to seven participants per group. Three key themes emerged: (1) sound as ‘Noise’ vs. ‘Music’; (2) acceptance of sound; and (3) coping strategies to manage unwanted sound (Figure 3). Interestingly, participants often gave examples of outdoor noise when explaining their perspectives, and as previously noted, people typically transition between indoor environments via outdoor environments.

#### 3.4.1 Key Theme 1: ‘Noise’ Versus ‘Music’

Participants considered that the same sound can be described as ‘noise’ or ‘music’ by different individuals, with participants indicating that ‘noise’ was considered unwanted sound, whereas ‘music’ was thought to be wanted sound. Classification of sound as either ‘noise’ or ‘music’ was seen to be dependent on the qualities of the sound, the health of the listener and the familiarity of the sound to the listener.

##### 3.4.1.1 Qualities of Sound

Qualities of sounds included the duration of the sound, volume, type of sound and ‘understandability’. Sound was considered to be ‘noise’ when it subjectively lasted what participants considered a longer or too long period, was at a higher volume, an unfamiliar or disliked type of song or sound, or could not be understood. In contrast, ‘music’ was considered what was subjectively described as a better duration, at an optimal or lower volume, a gentle type of sound, and could be understood in its context.

Volume appeared to have the greatest impact on whether a sound was categorised as ‘noise’ or ‘music’. When volume was greater than an optimal level for each individual, participants tended to use various onomatopoeic terms or phrases: ‘then *whoosh* a motorbike will go screaming past’ (Participant #A10), ‘the *boom boom boom* of the bass of the sound’ (Participant #A14) and ‘you'll hear this *wee‐ow wee‐ow wee‐ow wee‐ow* and that goes on and on and on’ (Participant #A8).

‘Understandability’ was primarily characterised as the ability to understand the words that are being presented within the sound, especially related to speech or song. When one was unable to understand the words, it was frustrating and felt more like ‘noise’ (Table 2).

**TABLE 2:** Key theme 1: ‘Noise’ vs. ‘Music’, identified via focus groups. Exemplary quotes for each of the subthemes: (a) qualities of sound, (b) health, (c) familiarity.

Qualities of sound	*It's a waste of my time because I can't understand a word they say. I can hear it. It's quite loud enough and its quite good volume but I can't distinguish the words they say*. (Participant #A13)
	*It's not always the sound, it's the comprehension of the sound that I find difficult at times. That you know there's the sound is there, you know something is happening – and I've done this with the television, and I have to turn it up a little because I'm not comprehending what is being said. I can hear it*. (Participant #A14)
Health	*Another thing I've noticed too is sitting in doctor's waiting rooms and they have the television on. And I think that in itself is hard to take if you're not feeling well and this sound is blaring out with a program that you really don't enjoy watching*. (Participant #A12)
	*Recently my husband, not long, passed from this world. And when he was very sick and he was a home … normal noise was absolutely important at that stage. … Normality was vital. And that noise outside was very welcomed*. (Participant #A14)
	*Well even what you are experiencing at the time, within your lifestyle – that can affect you. It may not be your health, but it may be your mental health, in that way*. (Participant#A14)
Familiarity	*As a child, I grew up being spoken to, and then I could respond. I wasn't allowed to speak until I was spoken to. And now, to me, people talking together is music and I love it. And I feel comfortable, no matter how loud it is. It's communication and I think that's great. So, language and communication within a group, to me, is very special no matter how loud it is*. (Participant#A14)
	*…that's a joy to hear the familiar old tunes and quite often we sing without music because, you know, we know it and it's comforting. It really is lovely. So, a familiar sound is really acceptable, and you welcome it*. (Participant #A14)

##### 3.4.1.2 Health

One's classification of sound as ‘noise’ or ‘music’ can be influenced by both physical and emotional health. Tolerance may decrease with physical illness or emotional fatigue, or mental ill‐health (Table 2). On the contrary, ‘normal’ noise could be reassuring or comforting, and lack of silence was welcomed by some when health was compromised.

##### 3.4.1.3 Familiarity and Previous Experience

Participants reported that familiarity or lack of familiarity affects how a sound is experienced. If familiar, past experiences will inform how it is now perceived, for better or worse. At the same time, absence of familiarity can also affect perception. For example, childhood experiences may inform how sound is perceived later in life (Table 2). Many participants compared sounds of the past with present‐day sounds. An example expressed by the participants was their dislike for ‘today's [popular] music’. Songs that the older adults grew up with were more familiar and reported as being better received (Table 2).

#### 3.4.2 Key Theme 2: Acceptance

Various factors emerged that potentially affected whether a sound was accepted by older adults, including the perception that the sound was essential or not. The impact of ageing and/or individual factors affected responses to sounds.

##### 3.4.2.1 Essential Versus Non‐Essential

One factor that affected the participants' acceptance of sound was whether it was considered ‘essential’ or not. These categories can be separated further into ‘necessary for self’ or ‘necessary for others’. For example, background music in shops/stores was perceived to be necessary for the proprietors to attract customers; it could be perceived as either necessary—for those who enjoy shopping to music—or unnecessary, for those who prefer ‘peace and quiet’ to concentrate on the task at hand. The potential relationship between background music and setting a mood was not raised. Traffic noise was considered necessary in terms of safety and assessing speed and proximity of vehicles. On the contrary, neighbours mowing the lawn was considered unavoidable for those who are actively mowing, but unnecessary for the non‐benefitting neighbour. Some participants felt that it was typically easier to tolerate different sounds if they were able to see the necessity of the sound, or if it was perceived to be shorter duration: ‘like the lawn mower man coming around or something like that, he's only going to be there for five minutes and then he's gone, you know what I mean?’ (Participant #D7).

##### 3.4.2.2 The Impact of Ageing

Many of the participants felt that as they aged they must simply accept the world around them, and that their changing needs were not necessarily addressed or catered for. This included noise levels within the community which they were not able to control, for example, shop/store owners setting background music volume levels at shopping centres. Hearing problems, noise sensitivity and the ability to concentrate were reported by participants as simply part of ageing, and therefore the participants felt like nothing could done about it. They had to try to accept and manage life around it. Some participants felt that their distaste for certain sounds was due to ageing, and therefore could not be changed. They felt that shops only catered for the younger generation (Table 3). A lot of the participants accepted their experiences of noise as a consequence of ageing. They felt that they were no longer catered for as they may not like some noises ‘just because we're getting old’.

**TABLE 3:** Key Theme 2: Acceptance, identified via focus groups. Exemplary quotes for each of the subthemes: (a) essential versus non‐essential sound, (b) the impact of ageing, (c) individuality.

Essential versus non‐essential	*I think a lot of it depends, too, on whether – you have to understand where the noise is coming from and why is it coming, you know what I mean? If it's something that could've been avoided, yeah it does annoy you. But if it's something that's happening, like the lawn mower man coming around or something like that, he's only going to be there for five minutes and then he's gone, you know what I mean?* (Participant #D7)
Impact of ageing	*And I wonder if a lot of accepting noise has to do with the brain function, I guess. And we are finding as we get older, [it's] harder to concentrate on two things at once, we're concentrating on what we're trying to do, but we're also trying to listen to something else*. (Participant #A14) *I think what turns me off is some of the shops you go into – the music is so loud that they probably cater for the younger group, of course, of which… you know*. (Participant #A10)
Individuality	*So, you don't know how people are going to accept [certain noise] ‐ the noise that we accept may not be acceptable to other people, but it may be*. (Participant #A14)

##### 3.4.2.3 Individuality

Several participants drew attention to the fact that all individuals have varying opinions on sound, noise and music. As one participant stated, ‘*Every person has an optimal rate of stimulation at which they perform best*’(Participant #A10). Individuals may feel that from their own individual experience, they must simply accept existing sounds which they cannot control without complaint (Table 3).

#### 3.4.3 Key Theme 3: Coping Strategies

A range of methods was described by older adults to manage their experienced effects of noise in the community. These include the adjustment of hearing aids, avoidance strategies and masking the sound with other sounds.

##### 3.4.3.1 Adjustment of Hearing Aids

Participants noted that hearing aid volume can generally be adjusted depending on the situation, for example, when background music at shopping centres is too loud:

[I have] a hearing aid. They're good. I've had them for a long time, and obviously they have a volume control, where I can switch out the background noise, or switch it on when I need it. (Participant #A9)

Participants also raised the importance of knowing when sound is needed and should not be ignored. For example, lowering the volume of hearing aids could be hazardous if one is unable to hear a truck speeding on the road. Some degree of sound perception is also needed to be aware of one's surroundings:

Another thing is it's so important to me. Noise is so important to me, I can't turn my head that way. And I need to know what's going over there. … So, it is important that I can hear what is on the other side of my line of vision. (Participant #A14)

##### 3.4.3.2 Avoidance Techniques

Techniques which participants mentioned using to avoid noise included online shopping and shopping at certain times of day. Although online shopping clearly avoids unwanted noise in shopping centres, some older adults reported not using it due to their unfamiliarity with using computers/internet, or concerns about financial security online:

Yes, we have. We have used it [online shopping] yeah, and that is good. I mean, I don't do it. My husband does it. I mean, I wouldn't know how to do it. (Participant #B5)

Can't do it. Won't touch online. Don't trust online, don't trust it with my money. (Participant #B3)

Multiple participants suggested that optimal times of the day for shopping are during school hours (10 AM to 2 PM) to avoid noisy school children and the associated busy period:

There are possibilities of going at a time when things are quieter… that, I think, is a big thing to avoid if the noise and confusion is stressful. You go at a different time and when there are not so many people. (Participant #B6)

##### 3.4.3.3 Masking Noise

When sound is unavoidable, it can be masked by other, more pleasant sounds, such as classical music:

That's right, beautiful classical music and this is it; I used to remember having a tape recorder beside my bed so that I didn't have to listen to the other sounds. I wanted to block those out. (Participant #B5)

## 4 Discussion

We initially hypothesised that older adults would be sensitive to noise and avoid certain locations due to noise. Our cohort of older adults often reported some sensitivity to noise, but using the assessment tools employed, scores for noise sensitivity were not markedly higher than studies of all aged adult cohorts (*n* = 1088, mean NS‐3 score 8.95) [43]. We found that our participants flagged common community locations as very noisy. Their self‐reported perception of noise was influenced by many factors, and a range of strategies was employed to cope with noise. Some participants reported avoiding locations altogether, some reported avoiding certain locations at certain times, others reported adjusting hearing aids in noisy locations, whereas other participants appreciated the value of masking unwanted sound with preferred music. The experience and acceptance of noisy environments appeared dependent upon multiple influencing factors: the qualities of the sound, the health of the listener, the familiarity of the sound to the listener, the perceived necessity of the sound, the understanding of individuality, beliefs around ageing and hearing aid use.

To date, there has been a dearth of literature exploring older adults' perceptions of noise and how it affects experiences of locations where older people may engage in the community for physical, emotional, cognitive and social benefits. This study focused primarily on the community‐based experiences of older community‐dwelling adults. It would be worthwhile to conduct further studies to consider older adults in additional residential or care settings, as well as in those with specific auditory health problems such as tinnitus or defined hearing problems, and other conditions potentially influencing noise sensitivity, such as dementia [45].

Our experience of the COVID‐19 pandemic highlighted potential risks of prolonged periods at home: social isolation, including physical inactivity, weight gain, behavioural addiction and inadequate sunlight exposure [46]. Furthermore, when considering social connectedness, older people often think about ‘getting out and about’ [2, p. 4], in the community, and community indoor environments are a crucial part of accessible everyday activities, providing opportunities for both physical activity and social engagement. For example, indoor environments can offer sheltered exercise and social engagement, as touted by mall walking groups who highlighted their protection from inclement weather [47]. Addressing noise exposure in this context is clearly a public health concern, and necessary if adults are to remain healthy, active and engaged as they age [1].

As the population in Australia, as elsewhere, enjoys greater life expectancy, it seems intuitive that community venues such as restaurants and shopping centres should be mindful of the experiences of older adults. Further research in this area has the potential to better inform ‘acceptable noise’ policies, and help develop training tools to assist with maintaining a quality environment for community end‐users. Acceptable noise standards, which take into account factors, such as overall level of noise, its frequency characteristics and its effect on both noise sensitive commercial and residential operations [48] may need revision to cater for the needs of older adults. We note that some participants flagged the potential for hearing aids to be used to adjust sound and noise perception. However, the potential advantages or disadvantages of, for example, turning off hearing aids to avoid unwanted noise were not raised, but would be interesting to explore. As participants noted, some sounds or noise are important to indicate danger. This is of relevance as technology changes‐ for example, modern electric cars have become quieter‐ so quiet that ‘artificial’ sound has been employed to warn pedestrians of their approach [49].

The potential economic implications of noise, and its modification, also warrant exploration, as both noise avoidance and the potential marketing benefits of enjoyable sound require study as our demographic patterns change. Organisations committed to conducting noise hazard awareness training in the workplace exist, but it may be useful to provide similar training tools to community providers who manage community venues commonly used by older adults. Noise considerations and data from studies such as our own may also inform choices when considering social prescribing for arts and health.

We acknowledge the limitations of this exploratory study, including limited sample size, and, as with any voluntary survey, selection bias is likely. This study was limited by recruitment of participants, with a 41% survey response rate despite a range of strategies used. This study occurred in a single independent living community which is not necessarily representative of other residential arrangements or communities. In modifying the NoiSeQ, we generated a non‐validated version, but we felt that this was a pragmatic approach, and reduced both research burden and aligned the questions more closely with our aims. We used numerical Likert scores to represent what is at heart a qualitative outcome, but this was practical for analysis and communication and, in this study, supplemented by qualitative data. The list of potentially noisy places in the community was not exhaustive, but few respondents submitted ‘other’ places beyond those listed. Although our focus was indoor noise, examples provided in focus groups often referenced noise generated outdoors, for example by vehicles, and we did not clarify if this was as experienced from indoor or outdoor settings. It would be interesting to investigate sociocultural factors and their potential influence on noise sensitivity, which we were underpowered to explore. We note that in a New Zealand study, ‘social deprivation’ was associated with increased propensity to noise sensitivity [50]. Furthermore, as the data were collected, hearing aid technology has advanced which may provide improved noise suppression algorithms in noisy environments. The uptake of online shopping has also increased, potentially impacting community shopping habits. Finally, although the respondents in this study were not working, many older adults may remain employed, and occupational noise should not be neglected for this group.

Although this study was exploratory, it provided new insights regarding noise sensitivity and experiences of older adults in relation to noise and coping strategies adopted. Multiple factors emerged as influencing the perception of noise, including sound quality, health of the listener, familiarity, impact of ageing, understanding of individuality, and the necessity of the sound. Larger‐scale studies are needed to explore noise burden and association with quality of life and other person experiences, in wider community and healthcare settings, as well as potential interventions to improve auditory and related experiences, in order to optimise experiences of older people and facilitate their remaining healthy, active and engaged members of the community.

## Consent

All participants provided written informed consent.

## Conflicts of Interest

The authors declare no conflicts of interest.

## Supporting information


**Appendix S1:** Survey.
**Appendix S2:** Semi‐structured interview guide for focus groups.
**Table S1:** Median and inter‐quartile range for Questions 4–18.

## Data Availability

The data that support the findings of this study are potentially available on request from the corresponding author and with additional Ethics Committee approval. The data are not publicly available due to privacy or ethical restrictions.
