# Supplementary material for: The Big Bang: How Does Indoor Noise Affect Lifestyle Choices of Older Community‐Dwelling Adults? An Exploratory Mixed Methods Study
Source: Australas J Ageing. 2026 Sep 7;45(3):e70226. doi: 10.1111/ajag.70226 (PMC13548338; doi:10.1111/ajag.70226)
Supplement: Supplementary file 1 [file AJAG-45-0-s001.docx]

**Supplementary Data**

Includes:

**Appendix 1:** Survey

**Appendix 2:** Semi-structured Interview Guide for Focus Groups

**Supplementary Methods**

Supplementary Results

**Supplementary Table 1**. Median and inter-quartile range for questions 4-18

**References for Supplementary Data**

**Appendix 1:** Survey. Note section 2 is derived from the 3-NS Noise Sensity Scale (3 questions, originally developed by Amman et al, 2007^1^), and section 3 modified from the NoiseQ scale (original by Shűtte et al, 2007^2,3^). Please see Supplementary Methods (below) and main text for additional information.

**Appendix 2:** Semi-structured Interview guide for focus groups

**Supplementary Methods**

*Noise sensitivity questions*

NS-3: good reported internal consistency and validity (Amman 2007).

NoiseQ: The original questionnaire consisted of 35 statements about noise in different environments while undergoing different activities which were rated from strongly disagree (1) to strongly agree (5). Questions considered less relevant to the target population of older adults and to our study aims, were modified/removed following stakeholder consultation (**Appendix 1**), leaving us with 18 questions. Changes to the original NoiSeQ included removal (n=17) and modification of the wording of three questions, namely, for ‘in a restaurant I cannot concentrate well on my conversation when people are talking loudly at other tables’ was modified to include: ‘restaurant/café/food court’, ‘On weekends I prefer quiet surroundings’ was amended to ‘In my free/private time, I prefer quiet surroundings’, and ‘I avoid leisure activities that are loud’ was modified to read ‘I avoid community meetings that are loud.’

*Focus group background: Literature review*

The literature review informing development of interview guide focussed on good health and well-being of older adults in the community, factors that affect how they make healthy lifestyle choices, impacts of noise on health, communication and noise as a falls risk, and regulation of noise in the community.

*Focus group participant identification*

Although individuals were present in person, name anonymity was maintained during focus groups by providing each participant with a badge number to replace use of first names.

*Inversion of Likert Scale scores*

Answers for questions 3, 7, 8, 11 and 16 on our questionnaire were inverted, for instance, 1 became 5, 2 became 4 and vice versa, due to the nature of the statement, in order to align with outcome of (positive) noise sensitivity

**Supplementary Table 1:** Median and inter-quartile range for questions 4-18 on our questionnaire (derived from the NoiseQ questionnaire, original by Schütte et al, 2007^1^). Likert scores for questions 7, 8, 11, 16 were inverted to align with ‘positive’ noise sensitivity.

| Q 4 | 4 (3-4) |
| --- | --- |
| Q 5 | 4 (3.25-4) |
| Q 6 | 4 (4-5) |
| Q 7 | 3 (2-4) |
| Q 8 | 4 (2-4) |
| Q 9 | 4 (3-5) |
| Q 10 | 4 (3-4) |
| Q 11 | 3 (3-4) |
| Q 12 | 4 (3-4) |
| Q 13 | 4 (3-4) |
| Q 14 | 4 (3-4) |
| Q 15 | 4 (4-4) |
| Q 16 | 3 (2-4) |
| Q 17 | 3 (3-4) |
| Q 18 | 4 (4-4) |

**References for Supplementary Data**

1. Amann E, Lercher P, Weichbold V, Eisenmann A. The effects of measuring noise sensitivity with a single or several rating scale on the population prevalence and distribution of this personality trait. Paper presented at the meeting of the Inter-Noise, Istanbul, Turkey, 2007. Accessed 26 November, 2024. Abstract at https://www.ingentaconnect.com/contentone/ince/incecp/2007/00002007/00000006/art00031

2. Schütte M, Marks A, Wenning E, Griefahn B. The development of the noise sensitivity questionnaire. *Noise Health*. 2007;9(34):15-24. doi:10.4103/1463-1741.34700

3. Schutte M, Sandrock S, Griefahn B. Factorial validity of the noise sensitivity questionnaire. *Noise Health*. 2007;9(37):96-100. doi:10.4103/1463-1741.37425
